# Radiographic and Symptomatic Knee Osteoarthritis 32 to 37 Years After Acute Anterior Cruciate Ligament Rupture

**DOI:** 10.1177/0363546520939897

**Published:** 2020-07-31

**Authors:** Joanna Kvist, Stephanie Filbay, Christer Andersson, Clare L. Ardern, Håkan Gauffin

**Affiliations:** † Unit of Physiotherapy, Department of Health, Medicine and Caring Sciences, Linköping University, Linköping, Sweden; ‡ Division of Physiotherapy, Department of Neurobiology, Care Sciences and Society, Karolinska Institute, Stockholm, Sweden; § Centre for Sport, Exercise and Osteoarthritis Research Versus Arthritis; Nuffield Department of Orthopaedics, Rheumatology & Musculoskeletal Sciences, University of Oxford, Oxford, UK; ‖ Department of Biomedical and Clinical Sciences, Linköping University, Linköping, Sweden; ¶ Sport and Exercise Medicine Research Centre, La Trobe University, Melbourne, Australia; Investigation performed at Medical Faculty of Linköping University, Linköping, Sweden

**Keywords:** ACL surgery, ACL repair, nonoperative management, radiographic osteoarthritis, symptomatic osteoarthritis

## Abstract

**Background:**

The long-term prevalence of knee osteoarthritis (OA) after anterior cruciate ligament (ACL) injury is unknown, especially in patients without a history of ACL surgery.

**Purpose:**

To (1) describe the prevalence of radiographic OA, symptomatic OA, and knee replacement surgery 32 to 37 years after acute ACL injury and to (2) compare the prevalence of radiographic OA, symptomatic OA, and knee symptoms between patients allocated to early ACL surgery or no ACL surgery and patients who crossed over to ACL surgery.

**Study Design:**

Cohort study; Level of evidence, 2.

**Methods:**

Participants aged 15 to 40 years at the time of ACL injury were allocated to surgical (augmented or nonaugmented ACL repair) or nonsurgical ACL treatment within 14 days of injury. At 32 to 37 years after the initial injury, 153 participants were followed up with plain weightbearing radiographs and completed 4 subscales from the Knee injury and Osteoarthritis Outcome Score (KOOS). Radiographic OA was defined as Kellgren and Lawrence grade 2 or higher. Symptomatic OA was defined as radiographic OA plus knee symptoms measured with the KOOS.

**Results:**

Participants allocated to ACL surgery (n = 64) underwent surgery at a mean ± SD of 5 ± 4 days (range, 0-11 days) after injury. Of the 89 participants allocated to no ACL surgery, 53 remained nonsurgically treated, 27 had ACL surgery within 2 years, and 9 had ACL surgery between 3 and 21 years after injury. In the total sample, 95 participants (62%) had radiographic tibiofemoral OA, including 11 (7%) who had knee replacement. The prevalence of radiographic tibiofemoral OA was lower in the group allocated to ACL surgery compared with the group who never had ACL surgery (50% vs 75%; *P* = .005). The prevalence of symptomatic OA (50% in the total sample) and patellofemoral radiographic OA (35% in the total sample) was similar between groups.

**Conclusion:**

Patients allocated to early ACL surgery, performed a mean 5 days after injury, had a lower prevalence of tibiofemoral radiographic OA at 32 to 37 years after injury compared with patients who never had ACL surgery. The prevalences of symptomatic OA, radiographic patellofemoral OA, and knee symptoms were similar irrespective of ACL treatment. Overall, the prevalence of OA after ACL injury was high.

**Registration:**

NCT03182647 (ClinicalTrials.gov identifier)

The odds of developing tibiofemoral joint osteoarthritis (OA) in the index knee after an anterior cruciate ligament (ACL) injury is 4 times higher than in the noninjured knee^2,34^ and 6 times higher compared with a noninjured population.^40^ A decade after ACL injury, up to half of patients have radiographic OA.^2,26,31,35^ In comparison, only 1 in 7 people of a similar age without history of ACL injury have OA.^44^ There is wide variability in the prevalence of patellofemoral joint OA after ACL injury, with a median of 50% at 10 to 15 years after ACL reconstruction.^11,24,33^ The odds of total knee replacement are 7-fold greater after ACL injury compared with a population without ACL injury.^22^

In research, knee OA is commonly defined based on radiographic findings (ie, radiographic OA). However, radiographic OA is poorly associated with patient symptoms.^9^ To better reflect outcomes that are important to patients,^39^ researchers use the term *symptomatic OA* to define a combination of radiographic OA and patient-reported symptoms. One definition of symptomatic OA is pain in the index knee during the previous 4 weeks plus evidence of radiographic OA (Kellgren and Lawrence scale score ≥2).^37^ Up to 1 in 3 patients experience symptomatic OA after ACL reconstruction more than 10 years after surgery.^32,33,35^

Development and progression of OA after ACL injury might differ depending on initial treatment. In a recent meta-analysis, the prevalence of radiographic knee OA was lower among people with nonsurgical treatment.^25^ Recurrent instability episodes after ACL injury may be associated with increased odds of medial meniscal damage,^41^ which is a risk factor for OA.^3,8,28,31^ However, most studies reporting an increased risk of meniscal damage in nonsurgically treated patients are retrospective reviews of patient records of patients subsequently undergoing reconstruction and exclude patients who have been successfully managed with rehabilitation.^25,41^ This highlights the need for further prospective research comparing rates of radiographic and symptomatic OA after surgical and nonsurgical management of ACL injury.

The prevalence of tibiofemoral OA increases with time from injury,^34,46^ irrespective of initial ACL treatment.^35^ However, the long-term prevalence of radiographic and symptomatic OA after ACL injury is unknown, especially in people without a history of ACL surgery.

Our study had 2 aims:

(1) To describe the prevalence of radiographic OA and symptomatic OA and the prevalence of knee replacement surgery 32 to 37 years after acute ACL injury
(2) To compare the prevalence of radiographic OA, symptomatic OA, and knee symptoms between patients allocated to early ACL surgery or no ACL surgery and patients who crossed over to ACL surgery

## Methods

This is a prospective cohort study. We followed 251 patients for 32 to 37 years after acute ACL rupture. At the time of their ACL injury, patients were aged between 15 and 40 years and received treatment at a university hospital (Linköping, Sweden) between November 1980 and December 1985. All patients who presented to the hospital emergency department with knee hemarthrosis had a knee examination under anesthesia and diagnostic arthroscopy. Concomitant meniscal or ligament injuries were treated based on severity (Table 1). Patients were allocated, according to year of birth, to surgical treatment (augmented or nonaugmented ACL repair) (even birth year) or nonsurgical ACL treatment (odd birth year).

**Table 1:** Baseline Characteristics, Concomitant Knee Injuries and Treatment, and Activity Levels Before and 4 Years After ACL Injury^*a*^

			Allocated to Nonsurgical ACL Management (n = 89; 58%)			
	Total Sample (N = 153)	Allocated to ACL Surgery (n = 64; 42%)	Total	Never Had ACL Surgery (n = 53; 60%)	ACL Surgery Within 2 Years of Index Injury (n = 27; 30%)	ACL Surgery >2 Years After Index Injury (n = 9; 10%)
Age at injury, y, mean ± SD	24 ± 6	24 ± 6	24 ± 6	25 ± 6	24 ± 5	19 ± 4
Sex, female	46 (30)	17 (27)	29 (33)	17 (32)	7 (26)	5 (56)
Preinjury Tegner Activity Scale score, median (minimum-maximum)	8 (3-10)^14^	9 (3-10)^3^	7.5 (3-10)^11^	8 (3-10)^4^	7 (4-10)^4^	8 (7-9)^3^
Concomitant meniscal injury	92 (60)	34 (53)	58 (65)	37 (70)	16 (59)	5 (56)
Surgically treated meniscal injuries	53 (35)	21 (33)	32 (36)	19 (36)	10 (37)	3 (30)
Concomitant cartilage injury	9 (6)	4 (6)	5 (6)	5 (9)	0 (0)	0 (0)
Surgically treated cartilage injuries, n	1	0	1	1	0	0
Concomitant MCL injuries	59 (39)	26 (41)	33 (37)	21 (40)	11 (41)	1 (11)
Surgically treated MCL injuries	35 (23)	17 (27)	18 (20)	10 (19)	7 (26)	1 (11)
Tegner at 4 y, median (minimum-maximum)	6 (0-10)^10^	7 (1-10)^3^	6 (0-10)^7^	6 (3-10)^3^	5 (1-7)^2*b*^	3 (0-6)^2^

a Data are presented as n (%) unless otherwise indicated. Superscript numbers indicate numbers of participants with missing data. ACL, anterior cruciate ligament; MCL, medial collateral ligament.

b *P* < .05 compared with the ACL surgery group.

The ACL was repaired with augmentation by use of the iliotibial band^5,30^ except in 15 patients who had ACL repair without augmentation. At the beginning of the study, nonaugmented repair was used only in patients with proximal ACL ruptures, but nonaugmented repair was abandoned in 1982 because at that time it was considered inferior to augmented repair. Augmented repair had been used initially for all midsubstance tears. The distal part of the torn ACL was repaired through use of pullout sutures. One bundle of the sutures was passed through a hole that had been drilled through the lateral femoral condyle at the site of the attachment of the ACL. The other bundle was passed over the top of the condyle where the 2 bundles were tied. In patients who had an augmentation, a distally based strip of the iliotibial band was used in addition to the repair. The strip was 1.5 cm wide and approximately 20 cm long. The strip was passed through the hole in the lateral femoral condyle anterior to the repaired ACL and then through a drilled hole in the tibia and secured to the anterior aspect of the tibia with a staple. This technique allowed a lateral tenodesis to be performed in addition to augmentation of the ACL. Results from different subgroups of patients at different follow-up time points have been presented previously.^5,28,30^

All patients completed structured rehabilitation: 4 to 6 months duration for patients with nonsurgical treatment, and 9 months duration after ACL surgery. After ACL surgery, the lower limb was immobilized for approximately 6 weeks in a long-leg cast, with the knee in 30° of flexion. Knee extension exercises were gradually increased.^5,30^

At 32 to 37 years of follow-up, we invited patients to complete a questionnaire, visit the movement laboratory at Linköping University for a clinical assessment of knee function, and undergo a radiological examination of both knees. A letter was sent to each patient regarding the follow-up procedure and included an informed consent form, the questionnaire, and reply-paid envelope. Up to 3 reminders were sent. Patients could provide informed consent to participate in 1 or more of the 3 study components (questionnaires, clinical assessment, and radiological examination). Ethical approval was granted by the regional ethical committee of Linköping (Dnr: 2017/119-31). This article presents results from the patients who provided consent for and attended the radiological examinations or had knee replacement surgery to the index knee.

### Outcome Measures

We used 4 subscales from the Knee injury and Osteoarthritis Outcome Score (KOOS) questionnaire (symptoms, pain, sports and recreation, and quality of life) to evaluate self-reported knee function. Each domain is scored out of a maximum 100 points, with a higher score indicating a superior outcome.^36^ We adapted previous KOOS criteria^14,17,18^ to classify participants as having knee symptoms, whereby participants who reported at least a 1-step decrease from the best response to at least 50% of items in the KOOS Pain and/or KOOS Symptoms subscale were categorized as having knee symptoms.

The single assessment numerical evaluation (SANE) was used as a global rating for each knee, whereby participants graded their right and left knees on a scale from 0 to 100, where 100 is the best (“If I had to give my knee a grade from 1 to 100, with 100 being the best, I would give my knee a ___.”).^38^ The Tegner Activity Scale was used to describe participants’ activity level before the injury, at 4 years of follow-up (data collected at that time), and at final follow-up.^43^ Participants reported the total number of knee surgeries to the index and nonindex knees.

OA in the tibiofemoral and patellofemoral joints was assessed by use of plain weightbearing radiographs. One radiologist, who was blinded to original treatment allocation, assessed all radiographs according to the Kellgren and Lawrence scale.^37^ The grading used was as follows:

- Grade 1: possible osteophytes
- Grade 2: definite osteophytes and possible joint space narrowing
- Grade 3: moderate osteophytes and/or definite narrowing
- Grade 4: large osteophytes, severe joint space narrowing, and/or bony sclerosis

We considered grade 2 or higher to be radiographic OA (see Appendix 1, available in the online version of this article).^37^ Knee replacement was scored as end-stage knee OA.^46^ Symptomatic OA was defined as radiographic OA plus knee symptoms (as defined above by use of the KOOS Pain and KOOS Symptoms subscales).

### Statistics

Mean and standard deviation or median and range were calculated for descriptive statistics. Comparisons between groups as allocated at baseline (ACL surgery and no ACL surgery) and as treated (ACL surgery, never had ACL surgery, crossed over to ACL surgery within 2 years, and crossed over to ACL surgery after 2 years) were made with analysis of variance with Bonferroni correction, Kruskal-Wallis tests, Pearson chi-square tests, and Fisher exact test, as appropriate.

## Results

Of 251 potentially eligible patients, 7 were deceased and contact details were missing for 10, leaving 234 as eligible to contact. A total of 4 patients declined to participate, 40 did not reply, and 190 participated in at least 1 of the 3 study components (response rate 81%). We excluded data from 6 participants: 1 had a new knee injury (tibial condyle fracture), 1 had rheumatoid arthritis, 3 had other generalized chronic musculoskeletal pain, and 1 had diagnostic arthroscopy more than 22 days after the index injury and did not meet the criteria for acute ACL injury. Further, 31 participants did not have radiograph examination at follow-up.

**Figure 1.**
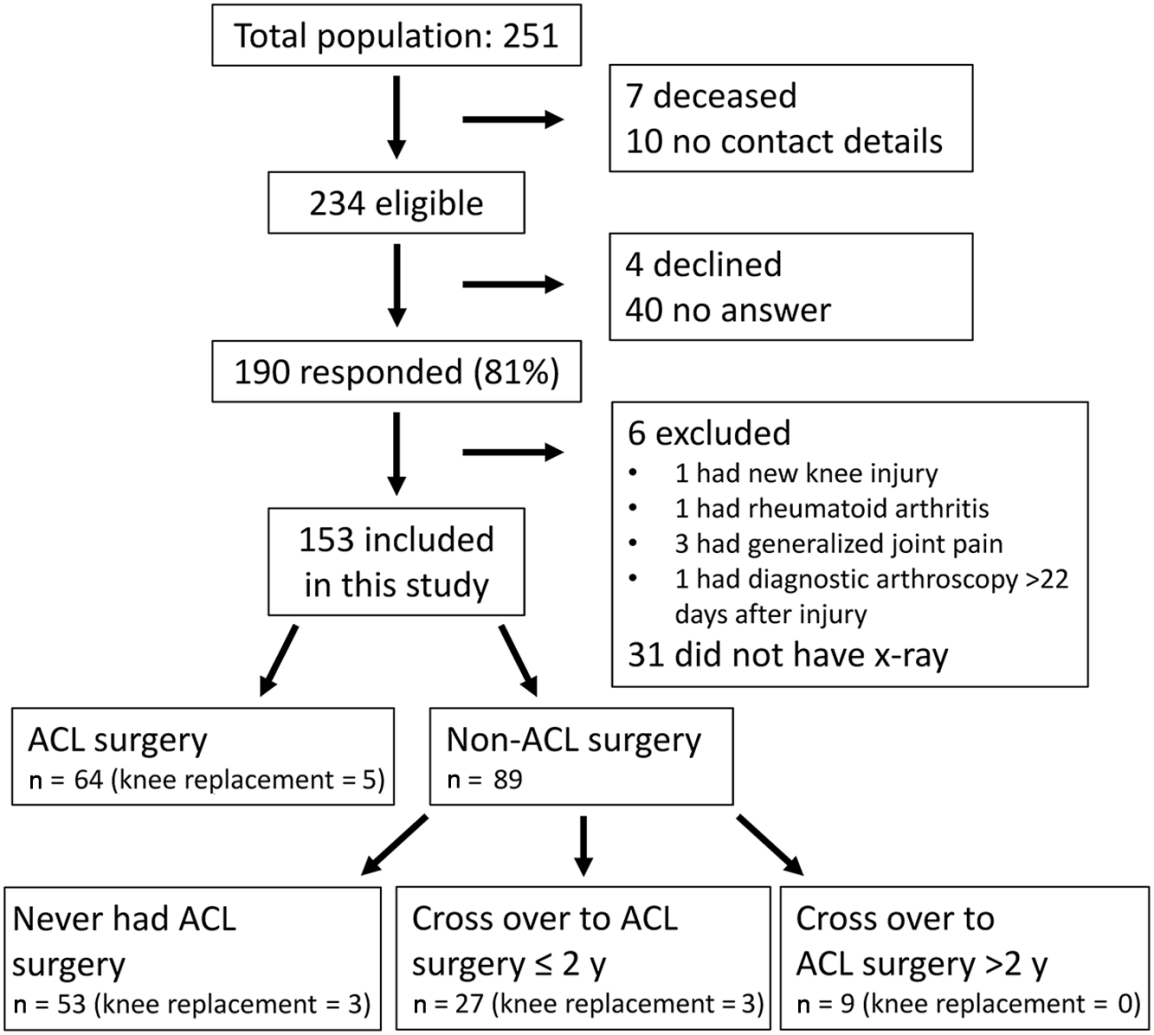
Flow of participants through the study. ACL, anterior cruciate ligament.

Data from 153 participants are included in this article: 142 participants who had radiographic examination and 11 participants who had knee replacement (Figure 1).

We found that 26 participants were incorrectly allocated after index injury: 9 participants with an odd birth year had ACL surgery, and 17 participants with an even birth year did not have ACL surgery. All participants had diagnostic arthroscopy at a mean ± SD of 5 ± 4 days after injury. The 64 participants allocated to ACL surgery had surgery at 5 ± 4 days (range, 0-11 days) after injury. Most participants (n = 46; 72%) had arthroscopy and ACL surgery the same day.

The 15 participants who had ACL repair without augmentation did not differ from the 48 who had repair with augmentation (regarding tibiofemoral radiographic OA or total number of surgeries during the follow-up period), and these participants were therefore analyzed as 1 group. A total of 89 participants were allocated to nonsurgical ACL management: 53 remained nonsurgically treated (never had ACL surgery), 27 had ACL surgery within 2 years after injury, and 9 had ACL surgery between 3 and 21 years after injury (Figure 1).

We found no significant differences in age, sex, body mass index at follow-up, preinjury activity level, concomitant injuries, total number of knee surgeries, or contralateral injuries between the ACL surgery group and the nonsurgical ACL treatment group or the individuals who crossed over to surgery (Tables 1 and 2). At 4 years of follow-up, the ACL surgery group had a higher activity level compared with the group that crossed over to surgery within 2 years (*P* = .01) (Table 1). No significant differences were found in self-reported outcomes (ie, KOOS or SANE, or knee symptoms as defined by KOOS) between the groups at 32- to 37-year follow-up (Table 2).

**Table 2:** Participant Characteristics and Outcomes at Final Follow-up (32-37 Years)^*a*^

			Allocated to Nonsurgical ACL Management (n = 89; 58%)			
	Total Sample (N = 153)	Allocated to ACL Surgery (n = 64; 42%)	Total	Never ACL Surgery (n = 53; 60%)	ACL Surgery Within 2 Years of Index Injury (n = 27; 30%)	ACL Surgery >2 Years After Index Injury (n = 9; 10%)
Age at follow-up, y	58 ± 6	59 ± 6	58 ± 6	58 ± 6	58 ± 6	53 ± 4
BMI at follow-up	27 ± 4	27 ± 4	27 ± 4	27 ± 5	27 ± 3	26 ± 3
Tegner Activity Scale score, median (minimum-maximum)	2 (1-7)	2 (1-7)	2 (1-7)	2 (1-7)	3 (1-7)	2 (1-3)
Total number of surgeries to index knee						
1 knee surgery	65 (45)	30 (49)	35 (42)	22 (47)	9 (33)	4 (44)
2 knee surgeries	40 (28)	16 (26)	24 (29)	15 (32)	8 (30)	1 (11)
>2 knee surgeries	39 (27)^9^	15 (25)^3^	24 (29)^6^	10 (21)^6^	10 (37)	4 (44)
Knee replacement surgery to index knee	11 (7)	5 (8)	6 (7)	3 (6)	3 (11)	0
Contralateral ACL injury	19 (12)	7 (10)	12 (14)	6 (11)	6 (22)	0
Knee replacement surgery to contralateral knee	4 (3)	1 (2)	3 (3)	2 (4)	1 (4)	0
KOOS Pain^*b*^	80 ± 19^1^	79 ± 19^1^	80 ± 19	80 ± 19	77 ± 21	84 ± 14
KOOS Symptoms^*b*^	69 ± 22^1^	66 ± 20^1^	71 ± 22	71 ± 20	70 ± 26	73 ± 26
KOOS Sports^*b*^	52 ± 28^2^	52 ± 28^1^	52 ± 28^1^	55 ± 26	46 ± 30	54 ± 34^1^
KOOS QoL^*b*^	54 ± 15^1^	53 ± 15^1^	55 ± 14	54 ± 13	56 ± 16	53 ± 15
SANE index^*b*^	69 ± 21	68 ± 20	69 ± 21	69 ± 18	69 ± 27	70 ± 24
SANE contralateral^*b*^	83 ± 20^16^	84 ± 19^7^	82 ± 21^9^	82 ± 19^7^	79 ± 25^1^	90 ± 13^1^
Knee symptoms^*c*^	102 (67)^1^	46 (73)^1^	56 (63)	33 (62)	17 (63)	6 (67)
ROA TFJ, index knee^*c*^	95 (62)	32 (50)	63 (71)	40 (75)^*d*^	17 (63)	6 (67)
ROA TFJ, contralateral knee^*c*^	43 (29)^3^	15 (24)^1^	28 (32)^2^	18 (35)^1^	10 (39)^1^	0
Symptomatic OA^*c*^	76 (50)^1^	28 (44)^1^	48 (54)	28 (53)	14 (52)	6 (67)
ROA PFJ, index knee^*b*^	48 (35)^15^	24 (42)^7^	24 (30)^8^	16 (33)^5^	6 (25)^3^	2 (22)
ROA PFJ, contralateral knee^*b*^	17 (12)	8 (13)	9 (11)	4 (8)	5 (20)	0
Combined TFJ and PFJ ROA, index knee^*c*^	46 (31)^4^	20 (32)^2^	26 (30)^2^	16 (31)^2^	8 (30)	2 (22)

a Values are expressed as mean ± SD or n (%) unless otherwise noted. Superscript numbers indicate numbers of participants with missing data. ACL, anterior cruciate ligament; BMI, body mass index; KOOS, Knee injury and Osteoarthritis Outcome Score; OA, osteoarthritis; PFJ, patellofemoral joint; QoL, Quality of Life; ROA, radiographic osteoarthritis; SANE, single assessment numerical evaluation; TFJ, tibiofemoral joint.

b Participants with knee replacement surgery are excluded.

c Participants with knee replacement surgery are included.

d *P* < .05 compared with the ACL surgery group.

### Radiographic and Symptomatic OA

**Figure 2.**
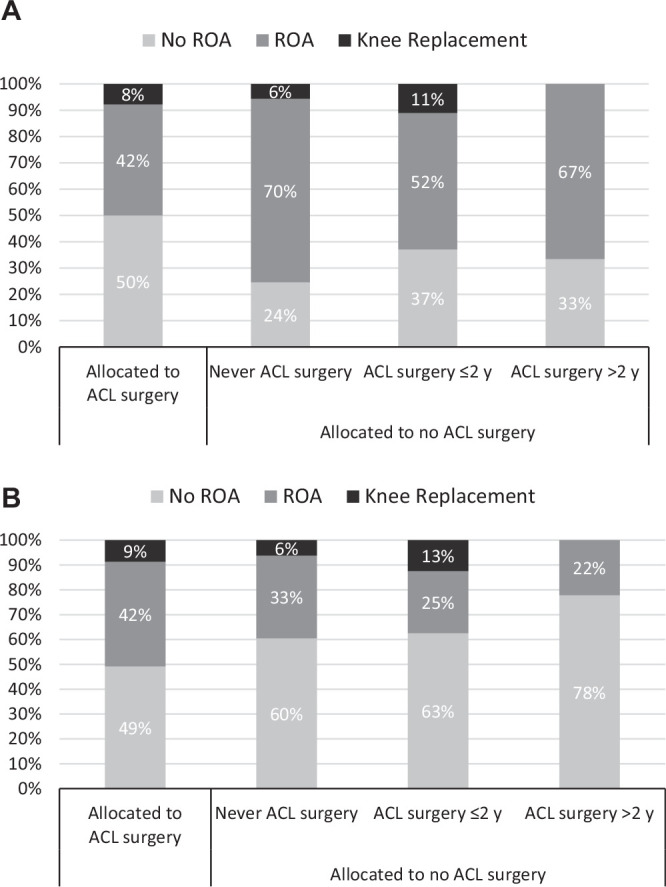
Distribution of participants with no radiographic osteoarthritis (no ROA), those with radiographic osteoarthritis (ROA), and those with knee replacement surgery in the (A) tibiofemoral joint and (B) patellofemoral joint. ACL, anterior cruciate ligament.

**Figure 3.**
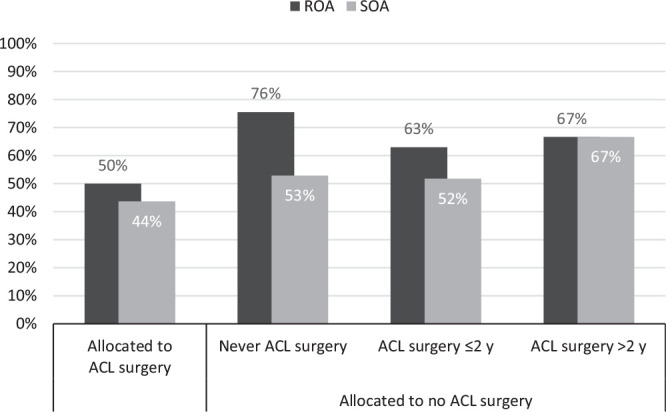
Distribution of participants with tibiofemoral radiographic osteoarthritis (ROA) including knee replacement and with symptomatic osteoarthritis (SOA). ACL, anterior cruciate ligament.

In total, 95 participants (62% of 153), including 11 participants (7% of 153) who had knee replacement surgery, had tibiofemoral radiographic OA. Further, 58 participants (38% of 153) had no tibiofemoral radiographic OA in the index knee. A lower prevalence of tibiofemoral radiographic OA was seen in the group allocated to ACL surgery (50% vs 75%; *P* = .005) compared with the group that never had ACL surgery (Table 2, Figure 2). No differences in symptomatic OA were seen between the groups (prevalence 50% in the study population; n = 153) (Table 2, Figure 3).

We found that 19 participants had sustained a contralateral ACL injury: 11 (58% of 19) had tibiofemoral radiographic OA in the contralateral knee, and none had contralateral knee replacement surgery. Of the 134 participants with no contralateral ACL injury, 32 (24% of 134) had tibiofemoral radiographic OA, including 4 (3% of 134) who had knee replacement surgery and 99 (76% of 134) who had no tibiofemoral radiographic OA in the contralateral knee (3 missing radiographs).

The prevalence of patellofemoral radiographic OA in the total population was 35%, with no significant difference between the groups (Table 2).

## Discussion

At 32 to 37 years after ACL injury, 62% of participants had tibiofemoral radiographic OA, including 7% who had knee replacement. Patients allocated to early ACL surgery (performed a mean 5 days after index injury) had a lower prevalence of tibiofemoral radiographic OA compared with patients who never had ACL surgery (50% vs 75%). Our results are different from previous studies, where there was no difference in the prevalence of radiographic OA between surgically and nonsurgically treated ACL injury.^10,24^

Despite higher tibiofemoral radiographic OA prevalence among patients who never had ACL surgery, patient-reported outcomes and symptomatic OA did not differ compared with patients who had ACL surgery. Our results support previous research.^10,19^ The image observed on radiographs is important to guide treatment if the patient has knee symptoms. However, radiographic findings often do not match the patient’s symptoms—a point that has been well-made by others.^9^ The implication for clinicians and patients when they are making treatment decisions is that radiograph-diagnosed OA alone is of insignificant clinical value.^24^ This point has important implications for researchers—it is insufficient to report on radiographic OA alone. Instead, researchers must focus on outcomes important to patients, including knee symptoms and patient-reported function,^9,39^ to help clinicians and patients make informed decisions.

### Previous Results From the Same Cohort

The outcomes at 32- to 37-year follow-up mirrored participants’ self-reported function^5,28,30^ and performance outcomes in earlier follow-ups of subgroups of our cohort (ie, 1.5, 4, and 15 years after initial injury), with 1 exception: At 15 years, a subgroup of our cohort (88 patients) had no significant difference in radiographic OA prevalence between the patients initially allocated to ACL surgery compared with the nonsurgically managed group. In the entire group, 50% had grade I or higher on the Ahlbäck score.^28^ Patients allocated to ACL surgery had less knee laxity at 1.5 and 4 years after initial injury,^5,30^ weaker quadriceps strength at 1.5 years,^30^ and similar quadriceps strength at 5 years^4^ compared with patients who received nonsurgical ACL treatment.

Patients in our study who had not had ACL surgery within the first 4 years after index injury had a higher rate of meniscal injuries at the short-term follow-up.^5^ Knee instability increases the risk for new meniscal injuries,^3^ and nonsurgical treatment may increase the requirement for subsequent meniscal surgery.^10^ Meniscal injury and pathology are predictors for future radiographic OA.^8,28,34^ Because we did not record new meniscal injuries in the total population after the 4-year follow-up,^5^ we cannot be sure of a possible effect of meniscal injuries on our OA prevalence results. However, no group differences were found in the total number of knee surgeries.

### Relationship Between Knee Surgery and OA

In our study, all patients received diagnostic arthroscopy, and almost three-quarters of the patients allocated to surgical treatment of the ACL had ACL surgery at the same time as the diagnostic arthroscopy. Therefore, all patients—irrespective of ACL treatment—were exposed to knee surgery. Knee surgery may increase the risk for tibiofemoral OA.^13^ Bleeding and inflammation initiated through the arthroscopy are hypothesized to predispose the knee to OA development.^15^ In our study, the biological features of patients’ knees were already altered due to the index knee trauma, so the negative effect of the arthroscopic procedure may have been reduced. In contrast, performing surgery soon after ACL injury may constitute a second trauma to the knee, resulting in prolonged elevation of synovial fluid levels of inflammatory cytokines, with potential to negatively affect healing of injured structures.^23^

Rehabilitation after injury or surgery may affect the development of OA. Patients who did not have ACL surgery started rehabilitation immediately and continued rehabilitation for 4 to 6 months. In contrast, after ACL surgery, all patients were immobilized for approximately 6 weeks in a long-leg cast. Postoperative immobilization can delay recovery of full range of motion after ACL reconstruction but does not negatively affect outcome at 2-year follow-up.^20^

### Timing of ACL Surgery and Predisposition to Knee OA

We noted that 27 patients (30%) crossed over to have ACL surgery within 2 years from injury, and 9 patients (10%) crossed over after 2 years. The latest primary ACL surgery was performed 21 years after the index injury. We chose 2 years as our threshold for early or late crossover because we expected that a decision for ACL surgery within 2 years from injury would be based on knee instability problems. We expected that ACL surgery performed later than 2 years after index injury might suggest that a new knee injury was sustained after a period of adequate knee function. We hypothesized that early or late crossover to ACL surgery may be associated with a different prevalence of OA because of different exposure to altered knee load that may arise due to chronic knee instability. In our study, approximately 1 in every 3 patients with ACL rupture initially treated nonsurgically needed surgical treatment of the ACL at some point after injury. Our results support previous studies.^19,29^

### Evolution of Approaches to Managing ACL Rupture

Long-term follow-up studies help to evaluate outcomes of treatment paradigms. Our study reflects the evolution of ACL injury treatment; primary ACL repair is not the contemporary approach to treating ACL injury, but it was in the 1980s^42^ when patients in our study received ACL treatment. So, our results may not be generalizable to current ACL reconstruction techniques. However, some new approaches to primary repair, including augmentation and bioenhanced repair,^27^ have preliminary results in selected populations.^1,21^ Primary ACL repair, with or without augmentation, can be performed only shortly after ACL injury. Patients in our study were allocated to have early ACL surgery (performed a mean 5 days after injury). The effect of timing of the surgery on patient-reported outcomes, meniscal or chondral pathology, or risk for OA is unclear.^3,8,12^

Approaches to postoperative rehabilitation have evolved in the 3 decades since our study commenced. The cast immobilization and weightbearing restrictions used when patients in our study received index treatment have been replaced by evidence-based programs that emphasize early progressive loading tailored to functional milestones.^45^ Different rehabilitation approaches may affect the risk for and prevalence of OA.

### Limitations

Our study has a long follow-up time and a high follow-up rate. However, knee OA and knee symptoms may be influenced by other factors unrelated to the ACL injury and treatment more than 3 decades ago. More than half of our patients had 2 or more surgeries to their ACL-injured knee. We do not have accurate data about the type of surgeries or the severity of subsequent knee injuries. Some patients had revision ACL reconstruction with various grafts (including bone–patellar tendon–bone, iliotibial band, or synthetic ligament) that may affect the risk of OA in different ways.^28^ Returning to sports after ACL injury increases the risk for new knee injuries^16^ and, subsequently, the risk for OA.^8,31^ We have data on sports participation at short-term follow-up (4 years after index injury), but we do not have data on sports participation between 5 and 30 years after the index injury. People change their preferences for activity participation after a knee injury sometimes because of impaired knee function but more often due to other priorities in life.^6,7^ Other life events, about which we do not have information, may have influenced the outcome. Because we did not adjust for confounding factors in our analyses, we cannot determine whether differences in OA rates between treatment groups are explained by other reasons aside from ACL treatment strategy.

## Conclusion

Patients allocated to early ACL surgery, performed a mean 5 days from injury, had a lower prevalence of radiographic tibiofemoral OA at 32 to 37 years after injury compared with patients who never had ACL surgery. The prevalence of symptomatic OA, and the patient-reported outcomes, including knee symptoms, function, and quality of life, were similar, irrespective of ACL treatment.

## Supplementary Material

Supplementary material
