# Supplementary material for: Radiographic and Symptomatic Knee Osteoarthritis 32 to 37 Years After Acute Anterior Cruciate Ligament Rupture
Source: Am J Sports Med. 2020 Jul 31;48(10):2387–94. doi: 10.1177/0363546520939897 (PMC7443961; doi:10.1177/0363546520939897)
Supplement: Supplementary material [file DS_10.1177_0363546520939897.pdf]

**Appendix 1:** Kellgren and Lawrence Scale classification for TFJ and PFJ

| | n=153 | Allocated to ACL surgery n=64 (42%) | Allocated to non-surgical ACL management n=89 (58%) | | | |
|---|---|---|---|---|---|---|
| | | | Total | Never ACL surgery n=53 (60%) | ACL surgery within 2 years of index injury n=27 (30%) | ACL surgery >2 years after index injury n=9 (10%) |
| ROA TFJ, index knee K&L Scale, n (%) | | | | | | |
| Grade 0 | 4 (3%) | 0 (0%) | 4 (5%) | 2 (4%) | 1 (4%) | 1 (11%) |
| Grade 1 | 54 (38%) | 32 (54%) | 22 (27%) | 11 (22%) | 9 (38%) | 2 (22%) |
| Grade 2 | 7 (5%) | 2 (3%) | 5 (6%) | 2 (4%) | 1 (4%) | 2 (22%) |
| Grade 3 | 8 (6%) | 3 (5%) | 5 (6%) | 2 (4%) | 1 (4%) | 2 (22%) |
| Grade 4 | 69 (49%) | 22 (37%) | 47 (57%) | 33 (66%) | 12 (50%) | 2 (22%) |
| ROA TFJ, contralateral knee K&L Scale, n (%) | | | | | | |
| Grade 0 | 34 (23%) | 14 (23%) | 20 (24%) | 8 (16%) | 6 (24%) | 6 (67%) |
| Grade 1 | 73 (50%) | 34 (55%) | 39 (46%) | 26 (52%) | 10 (40%) | 3 (33%) |
| Grade 2 | 4 (3%) | 2 (3%) | 2 (2%) | 1 (2%) | 1 (4%) | 0 (0%) |
| Grade 3 | 8 (6%) | 3 (5%) | 5 (6%) | 3 (6%) | 2 (8%) | 0 (0%) |
| Grade 4 | 27 (19%) | 9 (15%) | 18 (21%) | 12 (24%) | 6 (24%) | 0 (0%) |
| ROA PFJ, index knee K&L Scale, n (%) | | | | | | |
| Grade 0 | 8 (6%) | 2 (4%) | 6 (7%) | 3 (6%) | 2 (8%) | 1 (11%) |
| Grade 1 | 82 (59%) | 31 (54%) | 51 (63%) | 29 (60%) | 16 (67%) | 6 (67%) |
| Grade 2 | 14 (10%) | 10 (18%) | 4 (5%) | 3 (6%) | 0 (0%) | 1 (11%) |
| Grade 3 | 12 (9%) | 5 (9%) | 7 (9%) | 5 (10%) | 1 (4%) | 1 (11%) |
| Grade 4 | 22 (16%) | 9 (16%) | 13 (16%) | 8 (17%) | 5 (21%) | 0 (0%) |
| ROA PFJ, contralateral knee K&L Scale, n (%) | | | | | | |
| Grade 0 | 29 (20%) | 12 (20%) | 17 (21%) | 5 (10%) | 5 (20%) | 7 (78%) |
| Grade 1 | 96 (68%) | 40 (67%) | 56 (68%) | 39 (81%) | 15 (60%) | 2 (22%) |
| Grade 2 | 3 (2%) | 2 (3%) | 1 (1%) | 0 (0%) | 1 (4%) | 0 (0%) |
| Grade 3 | 7 (5%) | 1 (2%) | 6 (7%) | 4 (8%) | 2 (8%) | 0 (0%) |
| Grade 4 | 7 (5%) | 5 (8%) | 2 (2%) | 0 (0%) | 2 (4%) | 0 (0%) |

**Appendix 2:** Regarding the paper: "Radiographic and symptomatic knee osteoarthritis 32-37 years following acute ACL rupture"

Joanna Kvist, Håkan Gauffin, Stephanie Filbay, Christer Andersson, Clare Ardern, Lars Good, Magnus Odensten, Yelverton Tegner and Jack Lysholm

The importance of evaluating common orthopedic procedures and planning for long term follow-up examinations is unequivocal. As researchers, we are grateful to have had the opportunity to work with the late Professor Jan Gillquist, Linköping, Sweden. He created an atmosphere at Linköping University Hospital, Sweden, characterized by strict scientific thinking. Orthopaedic patients' pre-operative and post-operative outcome data were systematically registered, improved scoring systems to measure orthopaedic outcomes were developed[1-2] and meticulous follow-up examinations were routine between 1975-1995[3-5]. During that period, under the leadership of Professor Gillquist, Linköping University Hospital was at the forefront of innovation and research in arthroscopy, knee surgery and orthopaedic science. Professor Gillquist was both a pioneer in evidenced based medicine and in knee arthroscopy.
Knee arthroscopy was first introduced at Linköping University Hospital in the late 1970's and routinely performed throughout the 1980s, before this was adopted as the gold standard, globally.

It is an honor for us to publish 32-37 year follow-up data from one of the first studies comparing surgical and non-surgical treatment of acute anterior cruciate ligament injury. After finalizing that first randomized study in 1985[3], patients were continuously registered and followed up[4-6] as the clinical routine, enabling us to perform this long-term follow-up study with a large number of patients. In addition, we are grateful for the enthusiastic research-oriented team of orthopaedic surgeons and physiotherapists working at Linköping University Hospital in the 1980's, without the strong rapport they built with patients we would not have been able to achieve the exceptional follow-up rate of 81%, 32-37 years after acute ACL injury. Many patients wrote to us to express that they were grateful for the care they received at that time – both surgical and non-surgical interventions.

Although some aspects of anterior cruciate ligament injury management performed in the 1980s are now outdated (such as the use of postoperative plaster immobilization), other techniques used at Linköping University Hospital in the 1980s are regaining popularity amongst clinicians and researchers (including lateral tenodesis, ACL-repair and acute reconstructions). There is a lot to learn from a long-term follow-up study such as this. It provides unique insight into patient outcomes beyond 30 years of surgical and non-surgical management of ACL injury. It also demonstrates the history of many of today's evidence-based ACL injury management practices, as well as advancements in ACL management and the resurgence in popularity of historical practices for managing ACL injury. We owe previous researchers a lot, and we hope this work inspires current researchers to establish high quality longitudinal orthopaedic studies that future generations will benefit from.

**References**

1. Lysholm J, Gillquist J. Evaluation of knee ligament surgery results with special emphasis of the use of a scoring scale. Am J Sport Med 10. 150-154, 1982.

2. Tegner Y, Lysholm J. Rating systems in the evaluation of knee ligament injuries. *Clinical Orthopaedics & Related Research.* 1985(198):43-49.

3. Odensten M, Hamberg P, Nordin M, Lysholm J, Gillquist J. Surgical or conservative treatment of the acutely torn anterior cruciate ligament. A randomized study with short-term follow-up observations. *Clinical Orthopaedics & Related Research.* 1985(198):87-93.

4. Andersson C, Odensten M, Good L, Gillquist J. Surgical or non-surgical treatment of acute rupture of the anterior cruciate ligament. A randomized study with long-term follow-up. *J Bone Joint Surg Am.* 1989;71(7):965-974.

5. Andersson C, Odensten M, Gillquist J. Knee function after surgical or nonsurgical treatment of acute rupture of the anterior cruciate ligament: a randomized study with a long-term follow-up period. *Clin Orthop Relat Res.* 1991(264):255-263.

6. Meunier A, Odensten M, Good L. Long-term results after primary repair or non-surgical treatment of anterior cruciate ligament rupture: a randomized study with a 15-year follow- up. *Scand J Med Sci Sports.* 2007;17(3):230-237.
